# The parasite clearance curve

**DOI:** 10.1186/1475-2875-10-278

**Published:** 2011-09-22

**Authors:** NJ White

**Affiliations:** 1 Mahidol-Oxford Tropical Medicine Research Unit, Faculty of Tropical Medicine, Mahidol University, Bangkok, Thailand; 2 Centre for Tropical Medicine, Churchill Hospital, Oxford University, Oxford, UK

## Abstract

Parasite clearance rates are important measures of anti-malarial drug efficacy. They are particularly important in the assessment of artemisinin resistance. The slope of the log-linear segment in the middle of the parasite clearance curve has the least inter-individual variance and is the focus of therapeutic assessment. The factors affecting parasite clearance are reviewed. Methods of presentation and the approaches to analysis are discussed.

## Introduction

**Figure 1:**
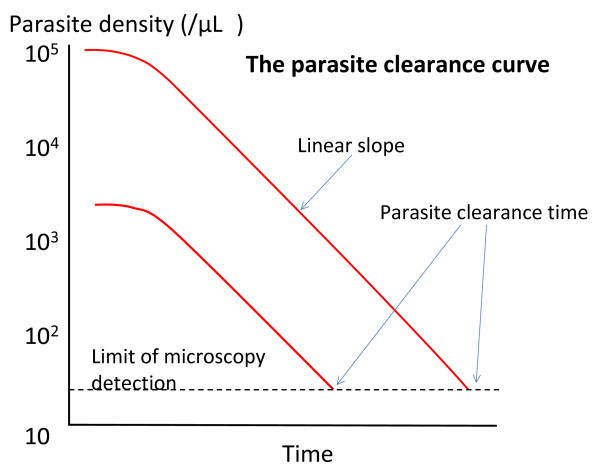
**Two *P. falciparum *parasite clearance curves with identical therapeutic responses illustrating the dependence of the parasite clearance time on pre-treatment parasite density**.

The objective of anti-malarial treatment is to cure the patient. A decline in malaria parasitaemia is essential for recovery from symptomatic malaria. Effective anti-malarial drugs given in the correct doses result a rapid decline in parasite densities. The parasite clearance time is the most frequently quoted measure of therapeutic response but it is an imprecise measure dependent on the pre-treatment parasitaemia. Most anti-malarials produce fractional reductions (parasite reduction ratios; PRR) in parasitaemia of between 100 and 10,000 per asexual cycle [1]. The graphic plot of the parasite densities that follow the start of anti-malarial treatment is commonly termed the parasite clearance curve (Figure 1). It is an important measure of the therapeutic response, particularly in assessing the artemisinin derivatives, which accelerate ring stage clearance. The factors which affect the parasite clearance curve are discussed and suggestions for presentation, analysis and interpretation are provided. Many of these factors interact with each other, and several are general issues related to parasite counting.

### Factors affecting the parasite clearance curve

#### Frequency of sampling

In most therapeutic assessments parasite counts are taken once daily initially, or only on days 2 and 3. This is insufficient for definition of individual parasite clearance profiles, although it is enough for therapeutic comparisons, particularly if sample sizes are large enough. To characterize parasite clearance profiles adequately at least four data points are required (i.e. counts at least twice daily), and to define lag phases adequately counts at ≤ 6 hour intervals are required. Counts are made until negative (usually either 200 or 500 white cells are counted on the thick film). Many investigators check a further slide 12 to 24 hours later to "make sure".

#### Estimating parasitaemia

Patients ill with acute falciparum malaria present with a range of parasitaemias. These initial parasite counts are approximately log-normally distributed. Counts vary over four orders of magnitude from approximately 100 to 1,000,000 parasitized erythrocytes/uL blood. In falciparum malaria, parasitized red cells circulate freely for only one third of the 48-hour asexual cycle. For the remainder they are sequestered in the venules and capillaries [2]. The peripheral blood parasite count is therefore a variable underestimate of the total parasite burden [2-4]. Parasitaemias in infections with *Plasmodium vivax, Plasmodium malariae*, and *Plasmodium ovale *are also approximately log-normally distributed, but very seldom exceed 100,000/uL. The zoonosis *Plasmodium knowlesi*, which has a quotidian cycle, may reach high parasite densities in humans and can be lethal. It is also not thought to sequester significantly. For these infections the peripheral blood parasite counts are an accurate reflection of the total burden. As parasite clearance is, for the most part, a first order process [5] then the higher the initial parasite density the longer counts will take to become undetectable (the parasite clearance time) (Figure 1).

**Figure 2:**
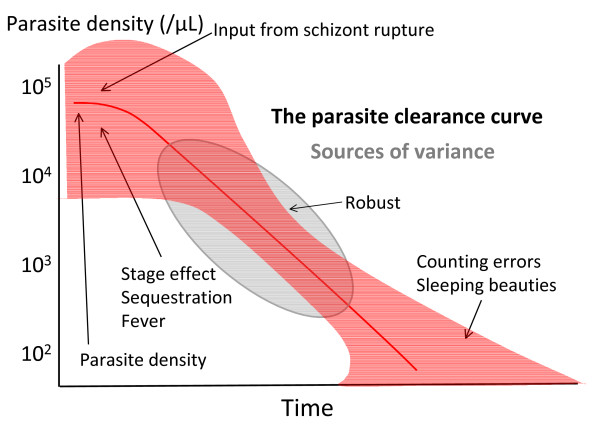
**Sources of variance in the *P. falciparum *parasite clearance curve**.

Counts at high densities (> 0.1% parasitaemia) are performed as the number of parasitized red cells per 1,000 red cells with multiple infected cells counted as a single unit [6]. As red cells are either cleared or pitted as a single unit this is not a significant source of error [7]. The parasite count is an estimate of the density of parasites circulating in the blood. There are always errors in this estimate, and these may be large at low parasite densities. These errors are both systematic and random [6]. Systematic errors are related to human error, excessive or inadequate blood on a slide, poor slide preparation, staining problems, the uneven spatial distribution of parasites within the thin blood film, and parasites being obscured or difficult to identify in thick films. Some people count more accurately than others so there may be both fixed and random errors associated with the person performing the count (hence the research investigations into automated counting and quantitative PCR methods) [8]. As thin film counts decline towards 1 per 1000 red cells the counts should switch to the thick film (0.1% parasitaemia approximates 100 parasites per 200 white cells on a thick film). In the thick film, parasites are counted either in a fixed volume of blood or per 100 to 1,000 (usually 200) white cells [9-13]. The fixed volume method seems slightly less precise. There has been debate whether to use a fixed white blood cell count of 8,000/uL or an observed count in calculating parasite densities, with both contributing to error [10,14,15]. White counts vary with age so using a fixed count leads to underestimates of parasitaemia in the young and overestimates in the old (11,14). In routine practice the fixed count method is more usual. In field assessments the thick film gives counts approximately 30% lower than the corresponding thin film [16]. At very high parasite densities estimation of parasitaemia from a thick film (when this was the only slide taken) is particularly inaccurate. In relation to the parasite clearance curve the transition from thin to thick film might therefore be expected to result in systematic error, but in an evaluation of approximately 4,000 serial parasite counts taken at ≤ 6 hourly intervals we have not found this to be the case, and so do not apply a correction. The change from thin to thick film, whichever method of thick film counting is used, does not affect the slope estimates (Stepniewska K: personal communication). Obviously at low parasite densities, close to the limit of detection, errors are greatest. There is often a tendency for microscopists to search a slide before starting the count resulting in counts of one parasite per unit number of white cells-thereby prolonging "clearance" and skewing the estimated slope of the linear portion of parasite clearance curve (Figure 2). Sometimes this "tail" to the parasite clearance curve is real. In some patients, particularly those who had initially high-density parasitaemias and were treated with artemisinin derivatives, low numbers of parasites (typically 1 per 200 to 1000 white cells) may persist for several days. This may represent persistency of dormant blood stages ("sleeping beauties"). Random errors are expected at low counts as parasite numbers vary in density even from a homogeneous sample in a Poisson distribution [17,18]. Thus errors are greatest at the beginning and the end of the parasite clearance curve. The most robust segment with the least inter-individual variance is in the middle of the curve, and the slope of this segment has therefore been the focus of assessment in therapeutic comparisons (Figure 2).

#### Stage of development; schizogony

The parasite population varies in age of development over the 48-hour cycle (24 hours for *P. knowlesi*, 72 hours for *P. malariae*). The asexual cycle begins at merozoite entry to the erythrocyte and ends at schizont rupture. Based on microscopy staging most symptomatic infections are unimodal in age distribution, but some may be bimodal or multimodal [4,19]. Developmental staging by microscopy is imprecise, providing age estimates within an approximate 4-6 hour time window. Whereas patients with severe malaria present with parasites at any mean age of development, in patients who present with uncomplicated falciparum malaria, young ring stages predominate in peripheral blood more frequently than would be expected by chance, i.e. random sampling of parasite age distributions. This well known observation, noted frequently in textbooks of tropical medicine, has been explained by synchronous schizogony resulting in a pulse release of pro-inflammatory cytokines, which provokes health-seeking behaviour [20]. If schizogony leading to schizont rupture is occurring at the time of presentation and start of treatment then new parasites will be entering the circulation rapidly as treatment is given and, if the rate and the numbers of new parasites entering exceeds the clearance rate, the parasite density will rise. If ingress to the circulation balances egress then counts remain unchanged. None of the available drugs prevent schizogony if mature schizonts are present. Thus abrupt rises in parasitaemia may occur just after starting treatment of synchronous infections [21-23]. Infections may comprise more than one parasite genotype, although one usually dominates, and usually derive from more than one sporozoite and thus hepatic schizont. Maturation and hepatic schizont rupture may not be synchronous. The clearance curve therefore represents a weighted average of the individual intra- host parasite populations' stages of development and clearances.

#### Stage of development; sequestration

In *Plasmodium falciparum *infections mature parasites (pigment containing trophozoites and schizonts) are seldom seen in the peripheral blood because they cytoadhere to vascular endothelium and so are sequestered [2]. This process begins at the large ring stage and may be accelerated by fever [24]. As a consequence parasitaemia can fall abruptly in a synchronous infection simply as a result of cytoadherence consequent upon extensive sequestration [3]. In such cases the predominant stages of parasite development in peripheral blood smears are large rings (i.e. the parasites which will soon sequester but have not yet done so) [4]. Equally, as parasites are released from the rupture of sequestered schizonts in falciparum malaria, parasitaemia may rise considerably, and to a much greater extent than seen in the other malarias which do not sequester, and are not therefore hidden from the microscope. The predominant stages of parasite development in peripheral blood smears are therefore tiny rings. Reduction in fever may result in an apparent slowing of initial parasite clearance by delaying the onset of sequestration.

#### Drug effects

Most anti-malarial drugs affect predominantly the more mature trophozoite stages of parasite development [25,26]. This results in clearance of the circulating parasitized erythrocytes in the so-called benign malarias, or in the case of falciparum malaria, death of the sequestered parasites [1]. As the sequestered parasites are not seen in the peripheral blood film the consequences of their killing on peripheral blood parasite counts are delayed. Furthermore unless the anti-malarial achieves therapeutic concentrations immediately (which occurs only with parenteral artesunate) there is also a variable delay related to drug absorption (which particularly slow for intramuscular artemether and artemotil) or slow intravenous infusion (required for quinine). As a result the changes in parasitaemia that occur immediately (i.e in the hours) after starting anti-malarial treatment are largely those that would have happened anyway [22]. Rises in parasitaemia result from schizogony and falls from sequestration [3]. In uncomplicated malaria parasite counts are seldom taken more frequently than once daily and these early fluctuations in parasitaemia may go unnoticed. In severe malaria following treatment with quinine, which does not affect greatly the circulating ring stages [27], the net result in an individual patient is that parasitaemia may, rise, fall or plateau until the parasites begin to sequester (an interval of no more than 12 hours) depending on the mean age and distribution of ages of the infecting parasites at presentation [28-31]. As a consequence the average profile in a patient series of parasite clearance times with quinine treatment is a brief down-sloping plateau followed by a log-linear fall. The sustained fall in parasitaemia results from some ring stage killing, but predominantly from the killing of sequestered parasites which cannot therefore mature and multiply to feed new young parasites into the circulation. For fully sensitive parasites chloroquine results in faster parasite clearance, which presumably reflects a broader stage-specificity of action compared with quinine [32].

**Figure 3:**
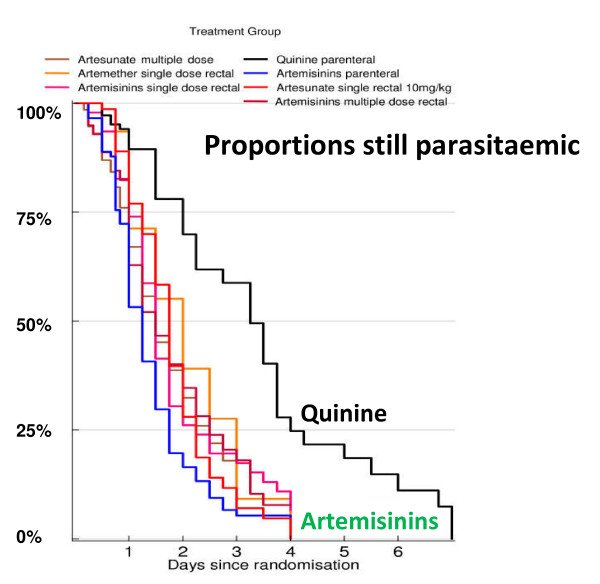
**The proportion of patients with falciparum malaria remaining parasitaemic after receiving rectal artemisinins or parenteral quinine in a series of randomized trials reviewed by Gomes *et al*, with permission **[47].

The pharmacological hallmark of the artemisinin derivatives is that they clear parasitaemia more rapidly than other drugs (Figure 3). In addition to this evident quantitative effect they alter the shape of the parasite clearance curve qualitatively [1,33] so that little or no initial plateau phase is evident, and parasitaemia starts to decline in a log-linear manner almost as soon as parasiticidal drug concentrations are reached. The slope of the log-linear segment of the parasite clearance curve is steeper with artemisinins than any other anti-malarial drug. This is explained by the killing of ring stage parasites, which are then "pitted" out of their host red cells and thereby removed from the circulation [34]. Artemisinin derivatives therefore prevent these parasites from maturing and sequestering [35]. This does not occur with quinine, which explains the life-saving benefit of artemisinin derivatives. The effects of artemisinin derivatives and quinine on sequestered parasites is estimated to be similar in-vivo [31]. The net result is a more rapid decline in parasitaemia with the artemisinin derivatives than any other anti-malarial drugs as the overall parasite killing per asexual life cycle is the product of the individual stage effects. The immediate rapid decline in parasitaemia is a consistent finding in all studies of the artemisinin derivatives where frequent parasite counts are taken (i.e. more than one count within the first 12 hours).

**Figure 4:**
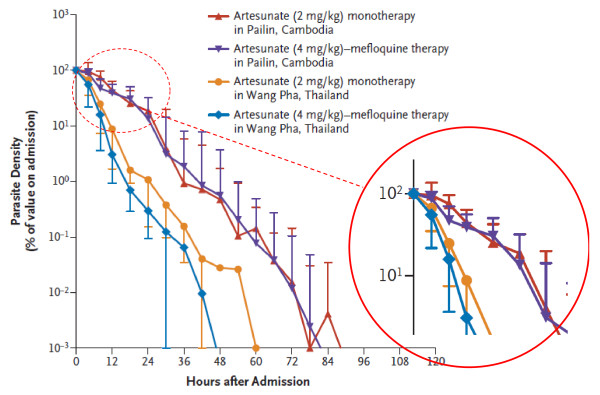
**Normalized *P. falciparum *parasite clearance curves showing the fraction of initial parasitaemia versus time in patients treated with artesunate in Western Cambodia and Western Thailand**. Parasite clearance was significantly slower in Western Cambodia. From Dondorp *et al*, with permission [37].

Artemisinin resistance has now emerged in *P. falciparum *in Western Cambodia [36,37]. This is manifest by marked slowing of parasite clearance. Unfortunately there is no ex-vivo test that reliably predicts this in-vivo phenotype so, until a marker is identified, assessments depend on detailed measurement of parasite clearance following observed treatment with quality assured artemisinin derivatives. The parasite clearance curves assessed in Western Cambodia were closer to those following quinine treatment of falciparum malaria (Figure 4). Conventional in-vitro testing suggested little difference in susceptibility between sensitive and resistant parasites [37]. This led to the suggestion that artemisinin resistance reflects a reduction in ring stage susceptibility, a proposition supported by mathematical modeling of pharmacokinetic- pharmacodynamic relationships [38].

#### Host defences

Haemoglobinopathies and glucose - 6-phosphate dehydrogenase deficiency may augment to host defences against malaria but the most important contributors are the non-specific and the specific immune responses to malaria. Therapeutic responses in malaria are enhanced by immunity [39]. Drugs which are clearly ineffective in non-immunes (usually children) may appear to be very effective in immune adults. Self-cure is usual in endemic areas even without anti-malarials. Antibody alone can be used to treat malaria [40,41]. Even in low transmission areas, with little background immunity, parasite clearance rates vary substantially between individuals infected with genetically identical parasites who receive artemisinin combination treatment [42]. This presumably reflects inter-individual differences in anti-malarial pharmacokinetics, splenic function and other non-specific host defences, and any acquired immunity. This remains difficult to characterize in malaria as there are still no good ex-vivo correlates of protective immunity [43]. Nevertheless some generalizations are possible. As immunity increases parasite counts are lower, severe malaria is less common, and parasite clearance is accelerated so the slopes of parasite clearance curves become steeper. In endemic areas this is reflected in the differing clinical presentations and therapeutic responses with increasing age. Conversely as immunity declines, for example if transmission is reduced, then parasite clearance rates fall.

### Presentation of data

**Figure 5:**
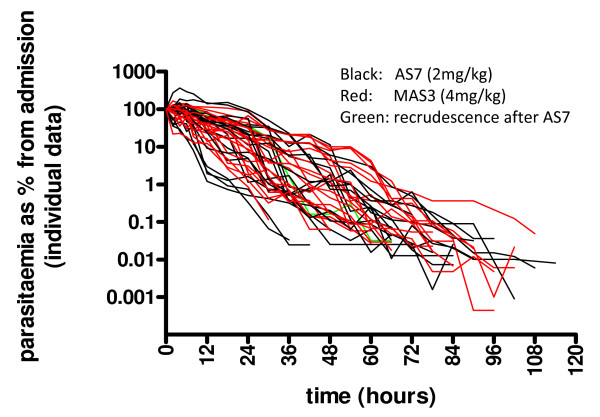
**Normalized raw data from the Cambodian study shown in Figure 4**.

**Figure 6:**
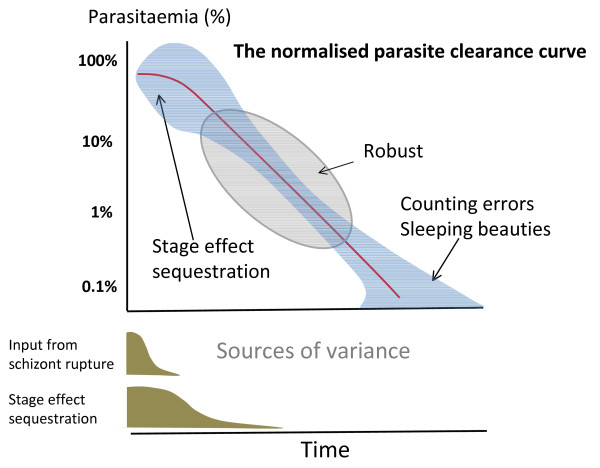
**The *P. falciparum *normalized parasite clearance curve**. Underneath the duration of variance derived from asynchronous schizont rupture and sequestration is shown.

Parasite clearance curves may be presented either as raw data (Figure 5), or summarized in a number of ways. The simplest is as mean and standard deviation values on a log scale, which shows the range of parasite densities but may obscure the contributions of stage specificity and sequestration. Normalization is justified if clearance rates are independent of densities (i.e first order kinetics and not Michaelis-Menten kinetics) (Figure 6). They generally are. However, high parasite densities are more likely in patients with little background immunity, so in studies conducted in areas of high transmission, a high parasitaemia may be cleared more slowly than a low parasitaemia simply because the patient lacks effective immunity against the particular infecting parasites, independent of any drug effects, but in areas of low transmission, where background immunity is lower, there is no evidence that parasite clearance within the range normally encountered in clinical trials is "capacity limited" (i.e. is saturable). Thus, in a series of patients normalization of parasite densities adjusts for their variance and is a valid approach. An alternative approach, which gives very little information about the initial changes in parasitaemia and therefore stage specificity of drug action, is to present the cumulative proportion of patients who are aparasitaemic by time (Figure 3)

### Analysis of data

Parasite clearance is usually analysed to assess the suitability of new treatments or to investigate changes in anti-malarial drug susceptibility. It is the drug effect (pharmacodynamics; PD) that is of primary interest, so the analysis attempts to account for the other confounders, which are drug-independent. Drug exposure (pharmacokinetics; PK) should also be assessed to determine how much of the observed variance in parasite clearance can be accounted for by inadequate drug exposure. PK-PD relationships for anti-malarial drugs are still poorly characterized, and most reported anti-malarial drug trials do not have a PK component, often leaving their interpretation uncertain. Parasite clearance has been expressed in a number of ways. The *parasite clearance time *(PCT) is most widely used, but this is a function of the pre-treatment parasite count (Figure 1), and it is critically dependent on the accuracy of microscopy and the frequency with which blood slides are taken. Any stage specificity of drug action is not reflected in this variable as PCTs are typically between 1 and 3 days. This may not matter in uncomplicated malaria but in infections with high parasitaemia and in severe malaria the speed of initial response is the critical determinant of outcome. The times taken for parasitaemia to fall by 50% (PC_50_) and 90% (PC_90_) have been used as alternatives, which are less affected by these covariates, but still require extrapolation from clearance curves [3]. The slope of the linear segment of the log parasitaemia versus time curve is a relatively robust variable as it is determined from a best fit to several data points well within the countable range, although it does not reflect fully stage specificity of drug action. The slope can be determined visually, using least squares regression, or by modelling. It remains to be decided what should be the criteria for defining the top of the slope (i.e. maximum value, or intersection of linear fit to the initial plateau, or modelled fit), and for the lowest densities that should be included at the tail of the curve [29].

### Thresholds

**Figure 7:**
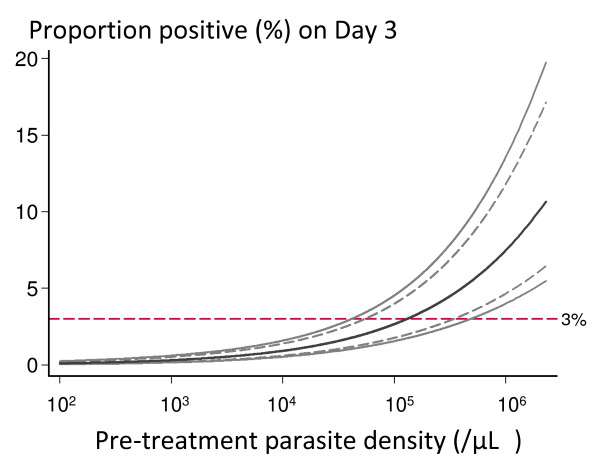
**The proportion of patients with fully artemisinin sensitive *P. falciparum *infections who are slide positive on day 3 are shown with 95 and 99% confidence intervals**. From Stepniewska et al with permission [45].

As parasite clearance is a function of host, parasite, and drug factors [1,44] there will always be considerable inter-individual variability in parasite clearance curves. In the wake of the discovery of artemisinin resistance in *P. falciparum *there has been considerable interest in devising threshold values for in-vivo monitoring studies, which could "rule-out" or "rule in" artemisinin resistance. Based on the largest available data set of in-vivo parasitological responses the malaria blood smear on Day 3 (72 hours) proved a good predictor of subsequent treatment failure and provides a simple screening measure for artemisinin resistance [45] (Figure 7). It was concluded that if "n" patients with parasitemias < 100,000/μl are given a currently recommended three day ACT the number with a positive day 3 smear should not exceed (n+36)/24; artemisinin resistance was considered highly unlikely if this proportion was less than 3%. The inclusion of a day 3 parasite count in routine studies provides a method for ruling out artemisinin resistance with a defined precision. Unfortunately, this has been misinterpreted to mean that if day 3 thresholds are exceeded then artemisinin resistance is present. It does not. It indicates the need for further study to characterize parasite clearance more accurately at the study location. In other words, the threshold "rules out" but it does not "rule in" artemisinin resistance. The day 3 value is also critically dependent upon precise timing of the samples - it should be a 72-hour value. Sometimes patients are treated in the afternoon and seen for follow-up in the mornings -in which case "day 3" is < 72 hours. Sometimes inclusive reckoning is practiced in which treatment starts on day 1, in which case day 3 is 48 hours not 72 hours.

## Conclusion

The parasite clearance time is a useful measure of anti-malarial drug effect but it is imprecise and depends upon the initial parasitaemia. More detailed assessment of the parasite clearance curve is needed to assess in-vivo responses to the artemisinin derivatives. Research is in progress to define the optimum methods and threshold values, which identify artemisinin resistance. Standardization of methodologies for the assessment of anti-malarial drug resistance is a primary objective of the WorldWide Antimalarial Resistance Network (WWARN) and a WWARN "parasite clearance estimator" providing a standard method of analysing parasite clearance data is now available as a freely accessible on-line tool [46]. This should facilitate comparison of in-vivo studies and help map the emergence and spread of resistance.

## Competing interests

The author declares that they have no competing interests.

## Authors' contributions

I wrote, read, and approved the final manuscript.
